# State Association Meeting a Great Success

**Published:** 1915-06

**Authors:** 


### State Association Meeting a Great Success.

The forty-ninth annual session of the State Medical Association, which convened in Fort Worth, May 4, 5, and 6, was one of the largest and most important conventions that the State Association has ever held since its organization. The Fort Worth committees had made every provision for the comfort and happiness of those attending the Association; they were very active and deserve a great deal of credit for handling in such a delightful and efficient way so large a gathering.

There were many prominent guests from other States, among them being the following: Dr. W. C. Rucker, Washington, D. C., assistant surgeon general of the United States Public Health Service; Dr. W. W. Grant of Denver, trustee of the American Medical Association; Dr. Oscar Dowling of New Orleans, president, and Dr. Seale Harris of Mobile, secretary of the Southern Medical Association; Dr. Fred J. Mayer of Opelousas, ex-president of the Louisiana State Medical Society; Drs. Albert H. Andrews and Joseph C. Beck of Chicago; Dr. Fred C. Zapffee of Chicago, secretary of the Association of American Medical Colleges; Drs. Isadore Dyer, F. W. Parham, S. M. D. Clark and L. Sexton of New Orleans; Drs. Henry W. Cook and William C. MacCarty of Rochester, Minn.; Dr. Edward H. Skinner of Kansas City; Dr. Frank A. Jones of Memphis, Tenn.; Dr. M. Smith, Oklahoma City, fraternal delegate from the Oklahoma State Medical Society and former councilor of the North Texas district of the State Medical Association of Texas; Dr. W. O. Talbot of Fort Worth, fraternal delegate from the Texas State Dental Association; Dr. A. O. Lundell, Fort Worth, bureau of animal industry, Department of Agriculture; John B. Hawley of Fort Worth, and Dr. William H. Deaderick of Hot Springs, Ark.

The official meeting place and exhibition rooms of the convention were at the Chamber of Commerce. Here out-of-town members registered and were given their credentials. The registration bureau, under direction of Dr. Holman Taylor, Secretary of the State Medical Association, had in conjunction with it a bureau of information. Miss Beulah Fulgham and Miss Susie Griffiths, who are experts, assisted Dr. Taylor.

A change was made in the by-laws and constitution of the Association, which provided for the naming of a president-elect. This, we believe, will prove very helpful, as it gives the president-elect one year to become acquainted with the workings of the Association before assuming his responsibilities as president.

Dr. J. M. Inge of Denton, Texas, has the honor to be the first president-elect of the State Medical Association; he will automatically succeed Dr. Moody; in effect, the Association named its 1915 and 1916 presidents at this meeting.

Newly elected officials for this year are as follows:

President—G. H. Moody, San Antonio.

President-elect—Dr. J. M. Inge, Denton.

Vice Presidents—W. F. Bledsoe, Port Arthur; H. C. Black, Waco; T. D. Frizzell, Quanah.

Trustee (re-elected)—W. R. Thompson, Fort Worth (five years).

Councillors—First District, Dr. F. P. Miller, El Paso (re-elected); Fourth District, Dr. S. C. Parsons, San Angelo (re-elected), Dr. C. C. Nash, Palestine; Thirteenth District, Dr. C. B. Williams, Mineral Wells; Fourteenth District, Dr. A. W. Carnes, Hillsboro.

Delegates to American Medical Association—Dr. A. B. Small, Dallas; Dr. Holman Taylor, Fort Worth; Dr. M. M. Carrick, Dallas.

### PRESIDENT AND SECRETARY COMMENDED.

Resolutions commending the efficient services of retiring President Frand D. Boyd and Secretary Holman Taylor were enthusiastically adopted.

### GALVESTON IS SELECTED.

Galveston was selected as the convention city in 1916 unanimously. The invitation was extended the doctors by Dr. W. C. Starley of that city, and his invitation was seconded by Dr. H. O. Sappington, who is one of Galveston's city commissioners. Telegrams and letters of invitation from the Galveston Chamber of Commerce, the Galveston County Medical Society and other prominent organizations were read by Dr. Starley.

The annual meeting of the State Association of County Secretaries was held Thursday at the First Baptist Church. Dr. W. H. Hargis was elected president; Dr. W. H. Blyth of Mt. Pleasant was re-elected vice president, and Dr. W. H. Wilder of Glen Rose was made secretary and treasurer.

The Roentgen Ray men met Tuesday morning at 9 o'clock in the Westbrook Hotel, Dr. Geo. D. Bond of Fort Worth presiding.

The memorial services held Tuesday night were very beautiful and impressive.

To Miss Hope Chase are due thanks for a most beautiful musical program. Dr. C. M. Rosser of Dallas and Dr. Fred J. Mayer of Opelousas, La., delivered impressive addresses to the memories of those who had died during last year.

Dr. Chase as chairman of the entertainment committee proved himself to be a past master in this art, for no meeting in the history of the Association provided more and varied entertainment than did the Fort Worth meeting.

Among the social features of the convention was the alumni banquets and receptions, particularly on Tuesday, which was devoted principally to "getting acquainted." An informal reception and tea was given Tuesday afternoon on the mezzanine floor of the Westbrook Hotel.

More than 200 doctors were the guests of Dr. Martin E. Taber of Dallas at a luncheon in the English room of the Westbrook Wednesday noon, given in honor of the medical men attending the State Medical Association convention from all sections of the country.

At noon Thursday the Association of Ex-Presidents was entertained at luncheon in the Westbrook by Dr. Bacon Saunders and Mrs. Saunders. At this luncheon Mrs. David Fly of Dallas and Mrs. F. E. Daniel of Austin, widows of former presidents of the State Association, were made honorary members of the Association of Ex-Presidents.

Officers were elected as follows: J. C. Loggins of Ennis, president; Bacon Saunders of Fort Worth, vice president, and Frank Paschal of San Antonio, vice president, and Holman Taylor, ex-officio secretary. Telegrams of felicitation were sent to members of the Association unable to attend, Drs. S. R. Burroughs of Buffalo, N. Y., and A. G. Clopton of Jefferson, Ill.

The cabaret dinner which followed the reception in honor of the State President, Dr. Boyd, was the main feature of the occasion. The show was held in the Coliseum on Wednesday night and consisted of a big banquet, music and vaudeville. It would be hard to make an estimate of the number of people who sat down to this banquet, but we remarked to one of our friends who stood near that we had never seen so many people eating at one time.

The social affairs for the visiting ladies was in charge of the following reception committee: Mrs. I. C. Chase, Mrs. Bacon Saunders, Mrs. Sidney J. Wilson, Mrs. S. A. Woodward, Mrs. Clay Johnson, and Mrs. J. H. McLean, and consisted of an informal reception on the mezzanine floor of the Westbrook Hotel Tuesday afternoon; Wednesday, 1 p. m., luncheon and reception for the visiting ladies was given in the ball room of the Metropolitan Hotel; 8. m., reception in honor of the President, Dr. Boyd, and wife. This was followed by the cabaret dinner.

On Thursday, 10 a. m., an automobile ride was given for the ladies; they were shown the entire city and the ride was concluded with a beautiful luncheon at the Country Club.

---

Recent statistics from the State Department show that only six counties out of the 250 have taken advantage of the Texas County

Hospital Law, passed so enthusiastically by the Thirty-third Legislature. It is the shame of Texas that willful ignorance of the benefits of these county hospitals has caused the defeat of numerous bond issues seeking to provide money for their establishment. These hospitals are open to the people of every class—the man who cannot afford medical treatment and the necessary operation is just as welcome as the rich man.

The mere statement that 24,000 people die in Texas every year from communicable diseases should be sufficient to convince any man of average intelligence of the worthiness of any movement, however high the cost, which would preserve the greater number of these sufferers to the State. A county hospital can be built at a very reasonable cost and maintained at a still more reasonable figure. Just why the people of any county should choose to turn down such a proposition, in the face of a statement of undeniable facts such as this, is incomprehensible—unless, indeed, we must confess that it is our ignorance that is responsible for our helplessness.—*Greenville Times.*

